# *Wohlfahrtiimonas chitiniclastica* Bacteremia Associated With Maggot‐Infested Ulcers and Tumor Lysis Syndrome in Ohio, USA: A Case Report

**DOI:** 10.1155/crdi/6760136

**Published:** 2026-07-29

**Authors:** Adil Menon, Morgan K. Morelli, Heather Hulec

**Affiliations:** ^1^ The MetroHealth System, Case Western Reserve University School of Medicine, Cleveland, Ohio, USA, case.edu

**Keywords:** bacteremia, case report, maggot-infested ulcer, MALDI-TOF MS, Ohio, *Tissierella praeacuta*, tumor lysis syndrome, *Wohlfahrtiimonas chitiniclastica*, zoonosis

## Abstract

**Background:**

*Wohlfahrtiimonas chitiniclastica* is an emerging Gram‐negative bacillus historically linked to larvae of the parasitic fly *Wohlfahrtia magnifica*. Although most human infections have been reported in Europe and Asia, cases are increasingly recognized in North America.

**Case Summary:**

A 74‐year‐old man from Ohio presented with sepsis and maggot‐infested decubitus ulcers. Blood cultures grew Gram‐negative, oxidase‐positive rods that could not be identified at the home institution. The isolate was referred to an outside hospital, where it was initially misidentified as *Edwardsiella* species before definitive identification as *W. chitiniclastica* by MALDI‐TOF MS (Bruker MALDI Biotyper), confirmed by supplementary biochemical testing, 6 days after the initial culture draw. Additional testing identified the rare anaerobe *Tissierella praeacuta*. Blood cultures at the home institution also identified *Proteus mirabilis* and *Staphylococcus aureus*. The clinical course was complicated by metastatic cancer and spontaneous tumor lysis syndrome (uric acid 14.6 mg/dL, LDH 2157 U/L) that improved following rasburicase therapy. The patient received empiric intravenous piperacillin–tazobactam for approximately 24 days, followed by de‐escalation to ceftriaxone. Follow‐up blood cultures were sterile. Goals of care were transitioned to palliation, and the patient died on hospital day 30.

**Conclusion:**

This case illustrates *W. chitiniclastica* bacteremia in Ohio, USA, and highlights diagnostic challenges including initial misidentification by conventional methods, the importance of MALDI‐TOF MS for definitive identification, and possible vector adaptation to regional muscid fly species. The coisolation of *T. praeacuta* underscores the complex wound microbiome associated with chronic neglected ulcers.

## 1. Introduction

*Wohlfahrtiimonas chitiniclastica* is an oxidase‐positive, nonmotile, Gram‐negative bacillus in the family Xanthomonadaceae, originally isolated from larvae of *W. magnifica*, a parasitic fly distributed across Europe, Asia, and North Africa [1]. Human infections typically arise in the context of myiasis and chronic wound colonization [2, 3]. While *Wohlfahrtia magnifica* is not endemic to North America, several recent U.S. cases suggest local adaptation involving nonparasitic muscid species, such as the common housefly (*Musca domestica*) and flesh flies (*Sarcophaga* spp.) [4–6]. We describe a case of *W. chitiniclastica* bacteremia in an elderly Ohio man with previously undiagnosed metastatic cancer, maggot‐infested ulcers, and tumor lysis syndrome (TLS). Notably, blood cultures also grew the rare obligate anaerobe *Tissierella praeacuta*, further illustrating the complex polymicrobial ecology of chronic neglected wounds [7, 8]. We discuss potential environmental vectors in the region and the diagnostic challenges posed by these unusual organisms. This case report was prepared in accordance with the CARE (CAse REport) guidelines; the completed CARE checklist is provided as Supporting Information 1.

## 2. Case Presentation

A 74‐year‐old man with failure to thrive and increasingly limited mobility was brought to the emergency department (ED) in Ohio after being found by a neighbor lying on the floor, incontinent with stool, and unable to rise. The patient reported being unable to ambulate for approximately 17 h and a decline in his overall health for the previous 4–6 months. He had not sought medical care for his entire adult life.

On presentation to the ED, his vital signs were as follows: BP 105/71 mmHg, pulse 111 bpm, temperature 97.6°F (36.4°C), respiratory rate 23, and SpO_2_ 93% on room air. He appeared ill, malnourished, and dehydrated, with multiple Stage IV decubitus ulcers over the sacral and gluteal regions, heavily colonized by maggots and necrotic tissue.

Laboratory evaluation showed leukocytosis (WBC 17 × 10^9^/L), lactate 4.8 ⟶ 4.2 mmol/L post 2 L IV fluids, hypoglycemia (59 ⟶ 43 mg/dL), hyperkalemia (6.4 mmol/L), HCO_3_^−^ 32 mmol/L, Cr 2.22 mg/dL, Ca 11 mg/dL, uric acid 14.6 mg/dL, and LDH 2157 U/L (with hemolysis). These findings were consistent with sepsis and spontaneous TLS in the context of metastatic malignancy [9, 10].

CT of the abdomen/pelvis revealed hepatic lesions, destructive pelvic masses, and pulmonary nodules consistent with metastatic malignancy of uncertain origin.

A summary of the clinical timeline is presented in Table 1.

**TABLE 1:** Clinical timeline.

Hospital day	Clinical event	Microbiological findings	Interventions
Day 0 (ED)	Presentation with sepsis, maggot‐infested Stage IV decubitus ulcers, failure to thrive	Blood cultures drawn	Empiric IV piperacillin–tazobactam initiated; IV fluids; rasburicase 6 mg IV for TLS
Day 1	Admitted to SDU; required HFNC, improved to room air	Blood cultures flagged positive: Gram‐negative, oxidase‐positive rods; isolate referred to outside laboratory	CT abdomen/pelvis: metastatic disease
Day 6	—	*W. chitiniclastica* identified by MALDI‐TOF MS (Bruker Biotyper) at outside laboratory	—
Day 11	Definitive identification communicated to treating team	Additional cultures: *P. mirabilis*, *S. aureus*, *T. praeacuta*	Surgical debridement of decubitus ulcers
Day 24	Clinical improvement	Follow‐up blood cultures: sterile	De‐escalation to ceftriaxone 1 g IV q24h; tube feeds initiated
Day 27	Transferred to general medicine floor	—	Multidisciplinary goals‐of‐care discussions
Day 29	—	—	Code status changed to DNR‐CC‐A‐DNI
Day 30	Increased somnolence, diminished respiratory effort; death	—	Comfort measures

Blood cultures were drawn in the ED, and empiric intravenous piperacillin–tazobactam was initiated. The patient was admitted to the step‐down unit (SDU) the following day (hospital day 1), where he initially required high‐flow nasal cannula (HFNC) for respiratory support but subsequently improved to room air.

Blood cultures flagged positive on hospital day 1, revealing small, Gram‐negative, oxidase‐positive rods. Definitive identification could not be achieved using the home institution’s conventional biochemical methods, and the isolate was referred to an outside hospital. The outside laboratory initially reported the organism as *Edwardsiella* species. However, this identification was inconsistent with the oxidase‐positive phenotype observed at the home institution, as *Edwardsiella* species are members of the Enterobacteriaceae and are characteristically oxidase‐negative [11]. This discrepancy prompted further investigation, and the isolate was subsequently identified as *W. chitiniclastica* by MALDI‐TOF MS using the Bruker MALDI Biotyper system 6 days after the initial culture draw [12, 13]. The identification was further confirmed by supporting biochemical testing, which demonstrated the characteristic phenotypic profile of *W. chitiniclastica*: oxidase‐positive, catalase‐positive, nonmotile, nonhemolytic, and nonfermentative [1, 3]. The definitive identification was communicated to the treating team approximately 11 days after admission. The isolate was susceptible to cefepime, meropenem, piperacillin–tazobactam, gentamicin, tobramycin, trimethoprim–sulfamethoxazole, and ciprofloxacin, consistent with prior reports of broad susceptibility [3, 14, 15].

Additional blood cultures grew mixed flora including *Proteus mirabilis*, *Staphylococcus aureus*, and *T. praeacuta*. *T. praeacuta* is a rare, obligate anaerobic bacterium within the phylum Firmicutes (class Tissierellia) that has been infrequently reported as a cause of human infection [7, 16]. Its isolation from blood cultures in this case is consistent with the polymicrobial, mixed aerobic–anaerobic bacteremia frequently observed in myiasis‐associated *W. chitiniclastica* infections and likely reflects translocation from the deeply necrotic, anaerobic wound environment of the patient’s Stage IV decubitus ulcers [17–19].

TLS was treated with rasburicase (6 mg intravenously once) and aggressive hydration, resulting in normalization of uric acid and LDH levels [9]. Surgical debridement was performed to remove necrotic tissue and larvae from the sacral and gluteal decubitus ulcers.

The patient remained on intravenous piperacillin–tazobactam for approximately 24 days. Following clinical improvement and clearance of bacteremia, antibiotics were de‐escalated to ceftriaxone 1 g intravenously every 24 h on hospital day 24. Follow‐up blood cultures obtained at that time were sterile, approximately 25 days after the initial culture draw. Urine cultures obtained concurrently suggested contamination. Tube feeds were initiated on hospital day 24 due to aspiration risk, as speech pathology evaluation determined that oral trials were premature.

By hospital day 27, the patient was clinically stable and was transferred from the SDU to the general medicine floor. Extensive multidisciplinary discussions were held among the family, palliative care, oncology, interventional radiology, and the primary team regarding available options. Cross‐sectional imaging had revealed metastatic disease of uncertain primary origin. After each specialty weighed in, the patient was not deemed a candidate for palliative chemotherapy, tissue biopsy was not recommended given his clinical status, and the consensus was to focus on comfort over aggressive treatment. The patient’s code status was changed to DNR‐CC‐A‐DNI on hospital day 29 after discussion with the family. On hospital day 30, the patient was noted to be more somnolent than on previous days with diminished respiratory effort. He was pronounced dead later that morning. The bacteremia had resolved with treatment, and death was attributed to the underlying metastatic malignancy. In total, the patient received approximately 24 days of piperacillin–tazobactam followed by approximately 6 days of ceftriaxone, for a total antibiotic duration of approximately 30 days.

## 3. Discussion

*W. chitiniclastica* is increasingly being recognized as a zoonotic pathogen capable of causing bacteremia and soft‐tissue infections, particularly in elderly or immunocompromised hosts [2, 3, 17]. Infection follows colonization of wounds by fly larvae acting as vectors or reservoirs [3]. In North America, potential vectors include *M. domestica*, *Lucilia sericata*, and *Sarcophaga* spp. [3, 4]. Given the patient’s chronic wounds and living conditions, inoculation via local muscid flies is the most plausible route.

The diagnostic trajectory in this case vividly illustrates the identification challenges posed by *W. chitiniclastica*. The isolate was initially misidentified as *Edwardsiella* species at the outside laboratory—a taxonomically implausible result given the fundamental biochemical discordance between the two genera. *Edwardsiella* species are oxidase‐negative members of the Enterobacteriaceae, whereas *W. chitiniclastica* is characteristically oxidase‐positive [1, 3, 11]. This misidentification likely reflects the absence of *W. chitiniclastica* from the reference databases of conventional biochemical identification systems [3, 15]. de Dios et al. similarly reported initial misidentification as *Acinetobacter lwoffii* by VITEK 2 before correct identification by MALDI‐TOF MS, and Bigge et al. demonstrated that VITEK 2 misidentified four of 21 *W. chitiniclastica* strains in their reference collection [20, 21]. Schröttner et al. emphasized that MALDI‐TOF MS or 16S rRNA gene sequencing is often required for definitive identification of this organism [3]. This sequence of events—spanning 6 days from culture draw to definitive identification, compounded by the need for referral to an outside institution—underscores the importance of correlating automated identification results with basic bench‐level biochemical tests such as the oxidase reaction and of maintaining a high index of suspicion for unusual Gram‐negative pathogens in the setting of myiasis‐associated wounds [3, 20, 21].

The polymicrobial nature of the bacteremia in this case—with coisolation of *P. mirabilis*, *S. aureus*, and *T. praeacuta*—is consistent with prior reports of polymicrobial bacteremia in myiasis‐associated *W. chitiniclastica* infections [17, 19]. Zhou et al. identified *W. chitiniclastica* alongside *P. mirabilis* and *Corynebacterium striatum* in a diabetic foot infection using metagenomic next‐generation sequencing, and Lysaght et al. described polymicrobial bacteremia including *Providencia stuartii* and *Ignatzschineria indica* in a patient with myiasis [19, 22]. El Hamzaoui et al. reported the first documented case of *W. chitiniclastica* bacteremia in Morocco, further illustrating the global reach of this emerging pathogen [23]. The coisolation of *T. praeacuta* in our case adds a notable dimension to this polymicrobial pattern. *T. praeacuta* is a rare, obligate anaerobic bacterium that has been infrequently reported as a cause of human infection; Caméléna et al. described only two cases—a septic pseudarthrosis and a bacteremia associated with pyonephrosis and hepatic abscess—and noted that MALDI‐TOF MS identification was inconclusive in both instances, requiring 16S rRNA gene sequencing for definitive identification [7]. The recovery of this obligate anaerobe from blood cultures in our patient likely reflects the deeply necrotic, hypoxic wound environment of the Stage IV decubitus ulcers, which creates an ecological niche favorable for anaerobic colonization and subsequent translocation into the bloodstream [18, 24]. Chronic wounds are known to harbor complex polymicrobial communities including obligate anaerobes that are frequently underestimated by conventional culture methods; 16S rRNA gene‐based analyses have revealed that fastidious anaerobic bacteria, including members of the Clostridiales family XI (to which Tissierella is related), are among the most prevalent taxa identified exclusively by molecular methods in chronic wounds [8, 16, 24]. The presence of *T. praeacuta* alongside the aerobic pathogens *W. chitiniclastica*, *P. mirabilis*, and *S. aureus* underscores the mixed aerobic–anaerobic ecology of these neglected wounds and the importance of anaerobic culture techniques in the microbiological workup of myiasis‐associated infections [18]. Notably, *T. praeacuta* is susceptible to beta‐lactams and metronidazole and was therefore covered by the empiric piperacillin–tazobactam regimen administered in this case [7].

In this patient, the coexistence of advanced malignancy and spontaneous TLS likely facilitated systemic invasion. Although rare in solid tumors, spontaneous TLS has been reported in association with metastatic disease, particularly with hepatic involvement [9, 10, 25]. Pettit and Liu found that spontaneous TLS in older adults with solid tumors carried a mortality rate of 74%, with renal failure and hyperphosphatemia (> 6 mg/dL) as strong predictors of death, and 88% of cases involved hepatic metastases [10]. Papapanou et al. similarly reported that liver metastases were significantly associated with TLS‐related death (OR 9.88; 95% CI 1.09–89.29) [25]. The prompt biochemical response to rasburicase in our patient underscores the importance of recognizing TLS beyond hematologic malignancies [9].

Antimicrobial susceptibility patterns of *W. chitiniclastica* remain broadly favorable, with susceptibility to beta‐lactam/beta‐lactamase inhibitor combinations, third‐generation cephalosporins, carbapenems, aminoglycosides, fluoroquinolones, and trimethoprim–sulfamethoxazole reported in most cases [3, 14, 15, 17]. However, Kopf et al. identified accessory genome‐encoded resistance determinants including beta‐lactamase (blaVEB), tetracycline, aminoglycoside, and sulfonamide resistance genes, suggesting that drug resistance may be expanding within the *W. chitiniclastica* clade and warrants ongoing surveillance [26]. In our patient, piperacillin–tazobactam was selected as empiric therapy for polymicrobial sepsis arising from chronically infected, necrotic wounds—a clinical scenario warranting broad‐spectrum coverage against Gram‐negative, Gram‐positive, and anaerobic organisms. The *W. chitiniclastica* isolate was confirmed susceptible, and the broad anaerobic activity of piperacillin–tazobactam made it particularly well suited for this mixed aerobic–anaerobic infection, providing effective coverage against all isolated organisms including *T. praeacuta* [7, 14]. De‐escalation to ceftriaxone—a third‐generation cephalosporin to which *W. chitiniclastica* is also typically susceptible—was performed after clinical improvement and sterile follow‐up cultures [3, 14, 15]. Surgical debridement was essential for source control and removal of the larval vector [2, 3].

From a bioethical standpoint, this case underscores the role of social vulnerability and neglected care in emerging zoonotic infections. The patient had not sought medical care for his entire adult life, and his chronic wounds and living conditions created the ecological niche for myiasis and subsequent bacteremia.

### 3.1. Limitations

Several limitations of this report should be acknowledged. Most notably, whole‐genome sequencing (WGS) of the *W. chitiniclastica* isolate was not performed. Because the isolate was referred to an outside institution for MALDI‐TOF MS identification and was not retained or returned for further analysis, genomic characterization—including phylogenetic placement, identification of antimicrobial resistance determinants, and assessment of virulence factors—could not be conducted. This is a significant limitation, as WGS has become an increasingly valuable tool for understanding the molecular epidemiology and evolutionary dynamics of *W. chitiniclastica* [15, 26]. Kopf et al. used comparative genomic analysis to identify accessory genome‐encoded resistance genes (blaVEB, tetracycline, aminoglycoside, and sulfonamide resistance determinants) and potential virulence traits across clinical and environmental isolates, demonstrating that WGS can reveal clinically relevant features not apparent from phenotypic susceptibility testing alone [26]. Similarly, Ren et al. employed WGS to characterize a clinical isolate from China, identifying novel genomic islands and contributing to the growing understanding of the species’ pathogenic potential [27]. The absence of WGS data in our case precludes determination of whether this Ohio isolate harbors unique resistance or virulence determinants and limits the ability to establish phylogeographic relationships with other North American strains.

Additionally, wound cultures from the decubitus ulcers were not obtained, which would have provided valuable information regarding the local wound microbiome and confirmed the presence of *W. chitiniclastica* and *T. praeacuta* at the primary site of inoculation. The exact timing of isolation of the copathogens (*P. mirabilis*, *S. aureus*, and *T. praeacuta*) relative to *W. chitiniclastica* was not precisely documented, and details regarding whether surgical debridement was performed in a single session or repeated were not available for review.

Despite these limitations, this case retains significant educational and scientific value. It expands the documented geographic range of *W. chitiniclastica* bacteremia to Ohio, contributing to the growing epidemiologic map of this emerging pathogen in North America [4–6]. The detailed diagnostic narrative—including the initial misidentification as *Edwardsiella*, the recognition of oxidase discordance as a critical clue, and the ultimate confirmation by the Bruker MALDI Biotyper with supplementary biochemical testing—provides a practical teaching framework for clinical microbiologists and infectious disease physicians who may encounter this rare organism [12, 13]. The coisolation of *T. praeacuta*, itself a rarely reported human pathogen, adds to the limited literature on this organism and illustrates the complex mixed aerobic–anaerobic ecology of chronic neglected wounds [7, 8, 24]. The clinical course, including the antibiotic management strategy with de‐escalation and the concurrent management of spontaneous TLS in the setting of metastatic malignancy, offers a comprehensive clinical reference. [9, 10, 25] Furthermore, the phenotypic susceptibility profile reported here adds to the cumulative body of antimicrobial susceptibility data that remains essential for guiding empiric therapy, particularly in resource‐limited settings where WGS is not readily available [3, 14]. The case also highlights the intersection of social determinants of health, neglected wound care, and emerging zoonotic infections—a theme of growing public health importance that transcends the molecular characterization of any single isolate.

## 4. Conclusion

This case expands the known geographic distribution of *W. chitiniclastica* infection, demonstrating its capacity to cause bacteremia in Ohio, USA, in association with maggot‐infested ulcers and malignancy. The initial misidentification as *Edwardsiella*—corrected only after recognition of oxidase discordance and subsequent MALDI‐TOF MS confirmation—underscores the diagnostic pitfalls inherent in relying on conventional biochemical identification systems for this emerging pathogen. The coisolation of the rare anaerobe *T. praeacuta* highlights the complex polymicrobial ecology of chronic neglected wounds and the importance of anaerobic culture techniques in the microbiological evaluation of myiasis‐associated infections. Adaptation to local muscid and calliphorid flies highlights the ecological flexibility of *W. chitiniclastica* beyond its original association with *W. magnifica*. Clinicians and clinical microbiologists should be vigilant for this organism in patients with chronic wounds, sepsis, and risk factors for myiasis, should correlate automated identification results with basic biochemical tests, and should ensure access to MALDI‐TOF MS for definitive identification when conventional methods fail. Future cases should prioritize isolate preservation and WGS to advance the molecular understanding of this emerging zoonotic pathogen.

## Author Contributions

Adil Menon conceptualized the study, performed microbiological interpretation, conducted the literature review, and drafted the manuscript. Morgan K. Morelli contributed to clinical data acquisition, patient care interpretation, and critical revision of the manuscript. Heather Hulec’s keen observation led to the initial identification of this unusual organism. Their persistence, meticulous attention to detail, and commitment to ensuring an accurate diagnosis were invaluable to the integrity of this study. Their contributions exemplify the importance of vigilance and perseverance in advancing scientific understanding.

## Funding

This work received no external funding.

## Disclosure

All authors approved the final manuscript.

## Ethics Statement

This case report was exempt from institutional review board (IRB) review in accordance with institutional policy for single deidentified case reports at the MetroHealth system. The patient is deceased; informed consent for publication was not obtainable. All patient information has been deidentified in accordance with the Health Insurance Portability and Accountability Act (HIPAA).

## Conflicts of Interest

The authors declare no conflicts of interest.

## Patient Perspective

The patient was critically ill throughout the hospitalization and died on hospital day 30. A patient perspective on the treatment received could not be obtained.

## Supporting Information

Additional supporting information can be found online in the Supporting Information section.

## Supporting information


**Supporting Information** Supporting Information 1: Completed CARE checklist.

## Data Availability

Data sharing is not applicable to this article as no datasets were generated or analyzed during the current study.
