# Supplementary material for: Wohlfahrtiimonas chitiniclastica Bacteremia Associated With Maggot‐Infested Ulcers and Tumor Lysis Syndrome in Ohio, USA: A Case Report
Source: Case Rep Infect Dis. 2026 Jul 29;2026:6760136. doi: 10.1155/crdi/6760136 (PMC13420265; doi:10.1155/crdi/6760136)
Supplement: Supplementary file 1 — Supporting Information Supporting Information 1: Completed CARE checklist. [file CRDI-2026-6760136-s001.pdf]

CARE
case report guidelines

CARE Checklist of information to include when writing a case report

(cc) BY-NC-ND

✓

Topic	Item	Checklist item description	Reported on Line
Title	1	The diagnosis or intervention of primary focus followed by the words "case report"	1
Key Words	2	2 to 5 key words that identify diagnoses or interventions in this case report, including "case report"	22
Abstract (no references)	3a	Introduction: What is unique about this case and what does it add to the scientific literature?	8-9
	3b	Main symptoms and/or important clinical findings	10-12
	3c	The main diagnoses, therapeutic interventions, and outcomes	12-17
	3d	Conclusion—What is the main "take-away" lesson(s) from this case?	18-21
Introduction	4	One or two paragraphs summarizing why this case is unique (**may include references**)	24-32
Patient Information	5a	De-identified patient specific information.	34-35
	5b	Primary concerns and symptoms of the patient.	35-37
	5c	Medical, family, and psycho-social history including relevant genetic information	37-38
	5d	Relevant past interventions with outcomes	38
Clinical Findings	6	Describe significant physical examination (PE) and important clinical findings.	40-46
Timeline	7	Historical and current information from this episode of care organized as a timeline	Table 1
Diagnostic Assessment	8a	Diagnostic testing (such as PE, laboratory testing, imaging, surveys).	43-46
	8b	Diagnostic challenges (such as access to testing, financial, or cultural)	54-58
	8c	Diagnosis (including other diagnoses considered)	46-48, 58-60, 63-66
	8d	Prognosis (such as staging in oncology) where applicable	80-84
Therapeutic Intervention	9a	Types of therapeutic intervention (such as pharmacologic, surgical, preventive, self-care)	50-51, 68-69, 70, 77
	9b	Administration of therapeutic intervention (such as dosage, strength, duration)	72-74, 68
	9c	Changes in therapeutic intervention (with rationale)	73-74,
Follow-up and Outcomes	10a	Clinician and patient-assessed outcomes (if available)	75-76, 86-88
	10b	Important follow-up diagnostic and other test results	75-76
	10c	Intervention adherence and tolerability (How was this assessed?)	72-74, 79
	10d	Adverse and unanticipated events	44-46, 77, 86-88
Discussion	11a	A scientific discussion of the strengths AND limitations associated with this case report	120-145
	11b	Discussion of the relevant medical literature **with references**.	90-119
	11c	The scientific rationale for any conclusions (including assessment of possible causes)	90-93, 94-100, 101-115, 116-119
	11d	The primary "take-away" lessons of this case report (without references) in a one paragraph conclusion	147-153
Patient Perspective	12	The patient should share their perspective in one to two paragraphs on the treatment(s) they received	163-164
Informed Consent	13	Did the patient give informed consent? Please provide if requested	Yes ☐ No ☒ Line: 156-158
